# Advances in the Study of Flame-Retardant Cellulose and Its Application in Polymers: A Review

**DOI:** 10.3390/polym17091249

**Published:** 2025-05-03

**Authors:** Quan Yuan, Shaodong Wang, Liping He, Shiwei Xu

**Affiliations:** 1 State Key Laboratory of Advanced Design and Manufacturing Technology for Vehicle, Hunan University, Changsha 410082, China; 2 College of Mechanical and Vehicle Engineering, Hunan University, Changsha 410082, China; 3 Suzhou Research Institute of Hunan University, Suzhou 215131, China

**Keywords:** cellulose, flame-retardant, modification, polymer

## Abstract

Cellulose, as a green and renewable polymer material, has attracted the attention of a wide range of scholars for its excellent mechanical strength, easy chemical modification and degradability. However, its flammability limits its application in automotive, aerospace, construction, textile and electronic fields. This review recapitulates the modification methods of flame-retardant cellulose and their applications in polymers in recent years. This paper discusses the fabrication of flame-retardant cellulose from various aspects such as boron, nitrogen, phosphorus, sulphur, inorganic and heterogeneous synergistic modification, respectively, and evaluates the flame retardancy of flame-retardant cellulose by means of thermogravimetry, cone calorimetry, limiting oxygen index, the vertical combustion of UL94, etc. Finally, it discusses the application of flame-retardant cellulose in actual composites, which fully reflects the extraordinary potential of flame-retardant cellulose for applications in polymers. Currently, flame-retardant cellulose has significantly improved its flame-retardant properties through multi-faceted modification strategies and has shown a broad application prospect in composite materials. However, interfacial compatibility, environmental protection and process optimisation are still the key directions for future research, and efficient, low-toxic and industrialised flame-retardant cellulose materials need to be realised through innovative design.

## 1. Introduction

**Figure 1:**
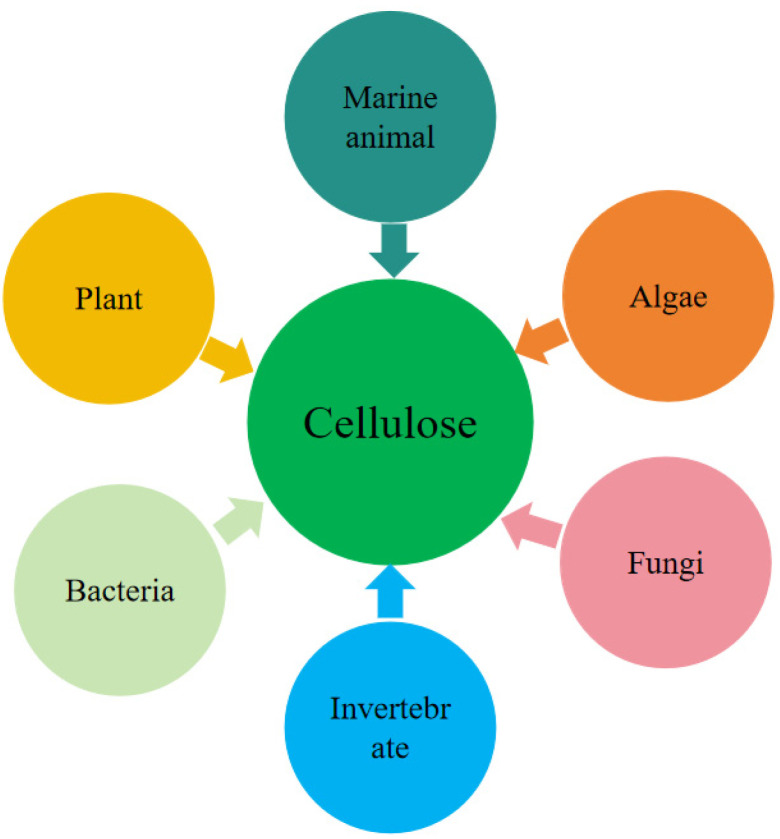
Major sources of cellulose.

Cellulose is one of the most abundant naturally occurring biomolecules in nature, is widely available and is one of the main components of the cell walls of most plants [1]. Cellulose can be derived from marine animals, algae, fungi, invertebrates and bacteria in addition to wood, hemp fibre, cotton, and bast fibres [2,3], shown in Figure 1. In this paper, we are discussing cellulosic materials mainly derived from the cell wall extraction of plant fibres. Cellulose morphologically exhibits a one-dimensional fibre shape with a high aspect ratio, but the specific morphological characteristics vary slightly depending on its source as well as processing and preparation techniques.

**Figure 2:**
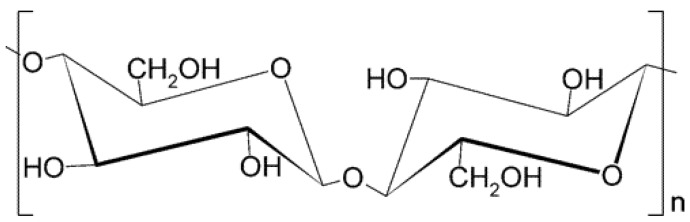
Chemical structure of cellulose.

Currently, cardboard and paper are the two most common cellulose-based materials. Under different preparation process conditions, cellulose can be converted into a wide variety of derivatives, which can be used to make some commercial products, such as cellophane and rayon [4], and the chemical structure of cellulose is shown in Figure 2. Since the discovery of cellulose, scholars at home and abroad have conducted a lot of research on the chemical composition and physical structure of cellulose. Cellulose is a polydisperse polymer formed by linking D-glucopyranose residues through β-1,4-glycosidic bonds, which consists of thousands of monomers condensed together, and the intrachain hydrogen bonding between the hydroxyl groups and oxygen of the linked ring molecules forms the linear configuration of the cellulose chain [5].

The chemical structure of cellulose is such that it burns easily and rapidly when exposed to open flames. The combustion process of cellulose is mainly composed of three stages: thermal initiation, thermal degradation and ignition. In the thermal initiation phase, cellulose undergoes phase and chemical changes. The degradation stage consists of two parts: the dehydration to carbon and the competitive reaction with levoglucose. Cellulose begins to undergo thermal degradation, which promotes the dehydration of cellulose to carbon; the ignition stage is the release of combustible gases and oxygen reactions to release a large amount of heat, which accelerates the combustion of cellulose [6,7]. The highly flammable characteristics of cellulose materials have severely restricted their large-scale application in fire prevention fields such as automobiles, high-speed railways and buildings. In recent years, the development of highly efficient, low-toxic and cellulose matrix-compatible flame-retardant systems has become a research focus in this field as the global demand for material sustainability and environmental compatibility increases.

Currently, the most common approach is to modify cellulose for flame retardancy through physical methods or chemical modification routes. Physical flame retardancy refers to the formation of an effective flame-retardant layer on the surface of cellulose by physical methods, such as coating, encapsulation, etc. Chemical flame retardancy refers to the introduction of new groups on the surface of cellulose through chemical reactions, thus endowing the cellulose with flame-retardant properties. The flame-retardant modification of natural cellulose-based materials cannot be separated from flame retardants, and the selection of suitable flame retardants or flame-retardant modifiers is the key to achieving their temperature and flame resistance. The flame-retardant mechanism of cellulose is generally divided into the condensed-phase flame-retardant mechanism, gas-phase flame-retardant mechanism and interrupted heat exchange flame-retardant mechanism [8].

Cellulose is the most abundant biopolymer in nature, derived from renewable resources such as plants and algae, which meets the global demand for green and low-carbon materials and has the advantages of renewability and sustainability compared with petroleum-based synthetic polymers. At the same time, the hydroxyl group (-OH) on the cellulose molecular chain provides an active site for chemical modification, which can be used to introduce flame-retardant groups through borate esterification, phosphorylation, nitrogen grafting and other reactions, to achieve highly efficient flame-retardant properties. This structural property makes cellulose more flexible and diversified in flame-retardant modification, and synthetic polymer flame retardants (such as halogenated compounds) are often accompanied by the release of toxic gases and persistent pollution, while cellulose-based flame-retardant materials can incorporate natural or low-toxicity modifiers (e.g., phytic acid, silica) to achieve an environmentally friendly flame retardant, reducing the hazards to the human body and the environment. Cellulose itself also has high mechanical strength, light weight and thermal stability, modified with flame retardancy and functions (such as thermal insulation and antibacterial). When used as a reinforcing filler in composite materials, it can simultaneously enhance the mechanical properties and flame retardancy of the matrix material, which is difficult to compare with synthetic polymers. Therefore, flame-retardant cellulose has a wide range of application prospects and can be used in automotive interiors, building insulation, electronic packaging, textiles and other fields, replacing traditional flame-retardant plastics and promoting the green transformation of the industrial sector.

Unlike the aforementioned reviews, which focused on synthetic polymers (e.g., epoxy resins, polypropylene) or general-purpose flame retardants (e.g., halogens, intumescent flame retardants), the previous reviews are broader in scope, focusing on traditional flame-retardant technologies (e.g., physical mixing, halogen flame retardant), mostly staying in the characterisation of flame-retardant properties, with less coverage of novel modification methods (e.g., nanocellulose, layer–layer deposition), and insufficient analysis of the relevance to the practical application scenarios. The limitations of cellulose flame retardancy (e.g., interfacial compatibility, durability) are usually only discussed in general terms in previous reviews. This review focuses on cellulose and its modification strategies and systematically analyses the mechanisms and effects of boron, nitrogen, phosphorus, sulphur, inorganic and synergistic modification, filling the gap of systematic review in the field of cellulose flame retardancy. It also discusses in detail the innovative technologies in recent years, such as phosphorus–nitrogen synergistic modification, metal–organic framework (MOF) composites and photo-initiated grafting; evaluates the effects of their application to composites (e.g., epoxy resin/cellulose composites, UL-94 V-0 rating of epoxy resin/cellulose composites, etc.), highlighting specific application cases of flame-retardant cellulose in functional materials (e.g., lithium ion battery gel electrolytes, waterborne polyurethane coatings) and analysing its synergistic effect in different polymer matrices (e.g., polypropylene, polylactic acid); and explicitly proposes the bottleneck of industrialisation (e.g., complexity of the process, high energy consumption), the demand for environmental protection (low-toxicity design) and long-term stability (water washing resistance validation) and other specific challenges, pointing out the direction for subsequent research.

Focusing on cellulose, this review not only highlights its unique advantages as a sustainable material but also distinguishes itself from previous broad reviews of non-combustible materials through systematic technical analysis and interdisciplinary application discussions. Its core value is to provide comprehensive theoretical support and practical reference for the scientific design and industrial application of flame-retardant cellulose modification.

## 2. Research Progress of Flame-Retardant Cellulose

### 2.1. Boron Flame Retardant Modification

Boron flame retardant is one of the commonly used flame retardants for cellulose flame retardant, mainly borax, boric acid (BA) and barium metaborate. Its flame-retardant mechanism is that when it is thermally decomposed, the boronic anhydride produced is encapsulated on the surface of the material to form a protective layer to isolate oxygen; at the same time, the cellulose in the combustion process is dehydrated, taking away a part of the heat, reducing the generation of combustible gases and having a flame-retardant effect [9].

Yang [10] added BA and borax to bamboo cellulose, which altered the thermal degradation process of the reaction and promoted the generation of residual carbon; the rate of heat release, total heat release and total smoke release were significantly reduced. Compared with the control group, the THR was reduced to 50.6% and 44.1%, and the TSR decreased by 95.3% and 91.6% after treatment with boric acid solution and borax solution, respectively. Zhang [11] used BA and its borates as additives, and the limiting oxygen index (LOI) of cellulose modified by a borosilicate binary system increased from 25.0% to about 30.0%, which showed good flame-retardant properties. Zhu et al. [12] prepared a phosphorus–boron and nitrogen synergistic flame retardant (rGBAP) using glycosyl-cross-linked boronic and phytanic acid (PA) and ammonium salts by a one-pot method, and the modified cellulose yielded an LOI value of 37.4%.

Wicklein [13] investigated the effect of BA and borate on the flame-retardant properties of carboxylated cellulose. The results showed that BA or borate reacts with the neighbouring dihydroxyl group at the 2,3 position in the cellulose glucose unit by complexation, and the complexation reaction is affected by pH; when pH = 10, the main cross-linking reaction is the formation of bis-chelate between the borate anion and the 2,3-diol on the cellulose glucose structural unit, while when pH is neutral, BA does not complex with the diols but may cross-link with the carboxyl group on the surface of the cellulose. Tong [14] firstly prepared cellulose by synergistically modifying it through quaternary ammonium salts and BA, which contained both N and B elements of flame-retardant cellulose. The flame retardancy was significantly improved by the boric acidification modification, and the LOI was up to 50.9% at a boron mass fraction of only 2.06%.

Cheng [15] used zinc borate (ZB)-modified cellulose to prepare aerogels. During the combustion process, the released H_2_O and ZnO gases diluted the oxygen concentration; meanwhile, ZB dehydrated to encapsulate an inorganic coating on the surface of cellulose, which effectively reduced its decomposition rate and heat release. Qin [16] prepared cellulose composite flame-retardant aerogels (WCF/ZB) by in situ synthesis. Zinc borate was uniformly distributed in the pores and on the surface of the aerogel. The zinc borate particles on the surface disappeared after the aerogel was burned, and a continuous, dense and swollen carbon layer was formed. Compared with the pure WCF aerogel, the HRR and the THR of the aerogel with the addition of 9% ZB solution were significantly decreased. The flame-retardant mechanism of WCF/ZB is that ZB produces a large amount of H_2_O at high temperatures, which carries away a large amount of heat on the one hand and dilutes the oxygen concentration on the other hand. In addition, ZB decomposes into ZnO and B_2_O_3_ after sufficient dehydration, forming an expanded carbon layer on the surface of cellulose, which not only inhibits the generation of combustible gases but also prevents the further degradation of cellulose.

Zhang [17] used commercially available bleached and unbleached (L) bamboo pulp cellulose as raw material, firstly phosphorylating bamboo cellulose modified by diammonium hydrogen phosphate/urea system (P) to prepare NFC (P/N FC and L-P/NFC), and then boronating them by borax (B) to produce flame-retardant NFC (B/P-NFC and L-B/P-NFC). The results showed that phosphate and boronic acid groups were successfully grafted on the NFC molecular chain. Phosphorylation improved the flame retardancy and residual carbon rate of NFC, and the pHRR was reduced by 40.5%. After boronation, the carbon residual rate increased to 2.89 times and the pHRR decreased by 83.58% to 30.17 W/g. Vertical combustion tests showed that both P/NFC and B/P-NFC had self-extinguishing phenomena.

These study cases (e.g., Yang’s treatment of bamboo cellulose with boric acid) did not assess the leaching risk of boron compounds during the service life of the wood (e.g., soil boron contamination due to rainfall washout) and did not propose a design solution for a low-mobility borate ester. Most of the experiments only tested the initial flame retardancy (e.g., LOI elevated to 50.9%) but did not simulate the effect of the actual use environment (e.g., high humidity, temperature cycling) on the stability of boron–cellulose bonding to prove its durability. Meanwhile, some of the sub-methods (e.g., zinc–boronic acid-modified cellulose aerogel) require a complex in situ synthesis process, which is energy-consuming and difficult to scale up, and the feasibility of simplifying the process (e.g., pre-mixing and dispersing) has not been explored.

### 2.2. Nitrogen Flame Retardant Modification

Nitrogen flame-retardant performance is more excellent, with low cost, low toxicity, smoke suppression, and good thermal stability to light, which under high-temperature conditions can generate NH_3_ and other gases, diluting the concentration of small molecules in the gas phase of combustibles and oxygen, and at the same time, the nitrogen flame-retardant thermal decomposition process will consume a part of the heat directly, thus achieving the purpose of the gas-phase flame retardant [9].

Meng [18] grafted acrylamide onto the surface of cellulose by a high-energy electron beam irradiation grafting method. The residual carbon rate was increased by 91% after grafting, and its maximum degradation rate was reduced from 2.35%/min to 1.33%/min. The results showed that the degradation rate of cellulose was slowed down and the residual carbon rate was increased after grafting modification, which helped to improve the flame-retardant properties of cellulose. Wang [19] prepared cellulose (CNF)/sodium montmorillonite (MMT) aerogels with excellent mechanical properties by modifying cellulose with melamine formaldehyde (MF) resin. The analysis showed that the introduction of MF promoted the formation of polymer protofibres in the connecting layer, and the limiting oxygen index value could reach 85%. Additionally, the UL-94 test was improved from flammable to V-0. Castellano [20] co-hydrolysed and co-condensed N-(phosphonomethyl)iminodiacetic acid generated from the modification of glycidoxypropyltriethoxysilane and tetraethyl orthosilicate (TEOS) to generate a novel phosphorus, nitrogen, and silica-containing non-halogenated flame retardant, which greatly improved the flame-retardant properties of cellulose.

**Figure 3:**
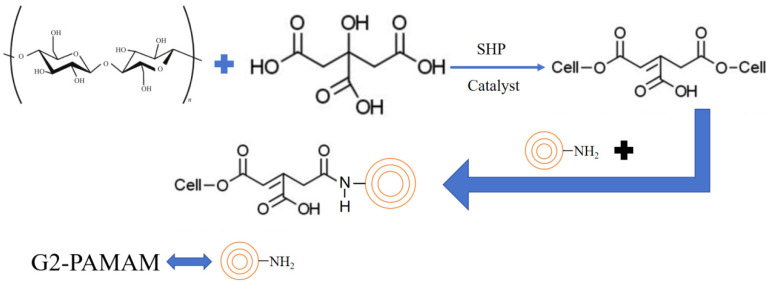
G2-PAMAM-modified cellulose process diagram.

Manfredi [21] found that for unmodified cellulose, the residual char and [CO_2_]/[CO] ratios tested by cone calorimetry (CCT) at 600 °C were about 12% and 39, respectively. This study also showed that cellulose burns rapidly in a short period of time with almost no residue. However, polyacrylamide (PAMAM)-impregnated cellulose had up to 30% residual carbon, and the [CO_2_]/[CO] ratio was only 9. Taherkhani and Hasanzadeh [22] found that cellulose treated with G2-PAMAM (second generation polyamide–amine dendritic polymer, as shown in Figure 3) also had high flame retardancy. Taherkhani and Hasanzadeh also investigated the flame retardancy of cellulose co-modified by citric acid with G2-PAMAM. The results showed that the treated cellulose had an LOI of about 23% and a residual char of about 25.1% compared to the control (18% and 1.2%). Vertical combustion tests showed that the control burned completely, whereas the char length of the treated cellulose fabric was only 0.32 cm. This demonstrates that the introduction of nitrogen-containing G2-PAMAM resulted in excellent flame-retardant properties of the treated cellulose.

The high temperature decomposition of nitrogen-based flame retardants (e.g., melamine formaldehyde resin) may release toxic gases such as HCN and NH_3_, e.g.. Wang et al. only focused on the LOI enhancement to 85% in their study and did not validate the safety by the flue gas toxicity test (e.g., FTIR analysis). Although the high-energy electron beam irradiation grafting method (e.g., Meng et al.) enhanced the residual carbon rate to 91%, the study did not evaluate its economic feasibility in industrial production lines because of the large investment in equipment (e.g., gas pedal cost) and high energy consumption. These nitrogen-based modification studies mostly focused on flame retardancy but did not explore their synergistic functions (e.g., antimicrobial, antistatic), limiting the potential for multi-scenario applications of modified cellulose.

### 2.3. Phosphorus Flame Retardant Modification

Phosphorus flame retardants, as one of the most widely used flame retardants, can be divided into inorganic phosphorus, organic phosphorus and phosphorus expansion flame retardants. Inorganic phosphorus flame retardant contains ammonium polyphosphate (APP), red phosphorus, ammonium phosphate, etc. This kind of phosphorus flame retardant is easily soluble in water and inexpensive. Organic phosphorus flame retardant mainly contains organophosphates, phosphate esters and phosphorus oxide flame retardants, with a high flame-retardant efficiency, high compatibility with cellulose and ecological and environmental protection, attracting the majority of scientific researchers’ attention [23].

The flame-retardant mechanism of phosphorus flame retardants includes condensed-phase flame-retardant and gas-phase flame-retardant mechanisms. When the flame retardant burns, it will decompose into phosphoric acid and polyphosphoric acid, which will polymerise to form metaphosphoric acid and polymetaphosphoric acid after dehydration, which will promote the dehydration of cellulose into carbon and form a char layer on the surface, thus hindering the transmission of oxygen and heat. At the same time, phosphorus flame retardants also decompose into some phosphorus-containing free radicals (e.g., ·HPO- and ·PO-), which inhibit and interrupt the reaction of the combustion chain segment by capturing flammable ·H- and ·OH- radicals in the gas phase, thus achieving a flame-retardant effect [9].

Guo [24] introduced an n-hydroxymethylphosphonic propionamide (MDPA) phosphorus-based flame retardant into cellulose to prepare gas-resistant aerosols. The results showed that MDPA was able to impart good flame-retardant properties to CNFs aerogels, with a significant increase in residual carbon yield from 0.15% to 40.37% and a significantly lower heat release rate.

**Figure 4:**
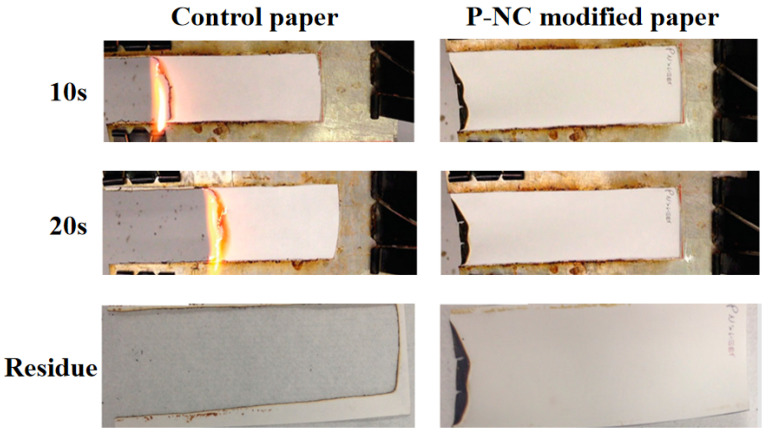
Combustion test of filter paper before and after modification.

Ghanadpour [25,26] successfully prepared high-performance flame-retardant cellulose with good self-extinguishing properties by the phosphorylation modification of cellulose using an NH_4_H_2_PO_2_/urea system, (shown in Figure 4). Yang [27] modified bamboo cellulose with phosphorus-based flame retardants such as Na_3_PO_4_-12H_2_O, NH_4_H_2_PO_2_ and APP and prepared flame-retardant bamboo cellulose by solution mixing. It was shown that the addition of flame retardants increased the amount of residual carbon and delayed the duration of combustion, which could effectively inhibit combustion. Similarly, Noguchi [28] successfully prepared phosphorylated cellulose by immersing coniferous wood pulp in an aqueous solution of diammonium hydrogen phosphate and urea as a raw material, and the phosphorylation did not lead to a change in the crystallinity of the cellulose. Recently, Kröger and Chen [29,30] modified cellulose fibres by phosphorylation using urea/phosphate and further optimised the process. This method was effective in preparing P-NC with better properties compared to the conventional method. In addition, Rol [31] used a twin-screw extruder to mechanically treat the phosphorylated cellulose to prepare P-NC with high solid content. The use of a twin-screw extruder to treat the phosphorylated cellulose significantly reduces the energy consumption in the preparation of P-NC and offers some possibilities for industrial production.

Blaine [32] modified flame-retardant cellulose with phosphoric acid, yielding higher thermal stability, and when it was coated on the surface of wood, the ignition time was greatly extended, and the dispersion of heat on the surface of the wood was promoted, showing excellent flame-retardant properties. Espinosa [33] successfully prepared CNC with phosphate on the surface by hydrolysing filter paper with phosphoric acid, and the CNC obtained had good thermal stability. Zhou [34] successfully prepared flame-retardant cellulose/aniline aerogels by doping H_3_PO_4_ and aniline, and the pHRR of the hybrid aerogel was only 33.4 W/g by CCT analysis, which was 73.2% lower than that of cellulose, and it could self-extinguish within 1 s.

Blilid [35] used cyclotriphosphonitrile-modified microcrystalline cellulose (MCC), and the thermal stability of the modified cyclotriphosphonitrile cellulose was substantially improved.

Marie [36] introduced dimethyl vinyl phosphonic acid into the cellulose molecular chain by a radiation method. The results showed that the phosphorus content in the modified cellulose was closely related to the high carbonation rate, which improved the carbonation of the cellulose and showed some self-extinguishing effects.

Zheng [37] synthesised novel 3-(hydroxyphenylphosphino)-propionic acid (3-HPP) cellulose esters using N,N-dimethylacetamide/LiCl as the raw material, and the results showed that the flame retardant 3-HPP reacted with cellulose to accelerate its dehydration into carbon and to reduce the release of combustible products.

Nguyen [38] synthesised two phosphorus-based flame retardants, diethyl 3-hydroxypropyl phosphoramidite (EHP) and 3-hydroxypropyldimethyl phosphoramidite (MHP), and it was found that the LOI of EHP/cellulose ranged from 25.8% to 33.4%, and that of MHP/cellulose ranged from 27.0% to 37.2% when EHP and MHP were added at a concentration ranging from 5% to 20%, respectively. With the increase in flame retardant content, the flame-retardant performance was significantly improved. This is because the char layer formed under the action of the flame retardant not only slows down the ignition of cellulose but also reduces the decomposition of cellulose. Moreover, the hydroxyl group at the end of amidophosphorus will catalyse its decomposition to generate an acidic intermediate, which reacts with cellulose to change its thermal decomposition process, thus improving the flame retardancy. In addition, the presence of short-chained alkoxy groups on MHP makes its flame retardancy effect better than that of EHP.

In addition, Sirvio [39] successfully grafted bisphosphonates onto the cellulose surface by using sodium periodate oxidation and the Schiff base reaction to prepare P-NC. TG analysis showed that P-NC had good thermal stability and char formation ability. Suo [40] designed and prepared a novel covalently modified CNC-based flame retardant (CNC@DPP) by the esterification reaction of CNC with diphenyl phosphate (DPP). The excess -OH in CNC@DPP was chelated with Zn^2+^ to prepare a CNC@DPP@Zn composite flame retardant, which could further reduce the smoke generation.

**Figure 5:**
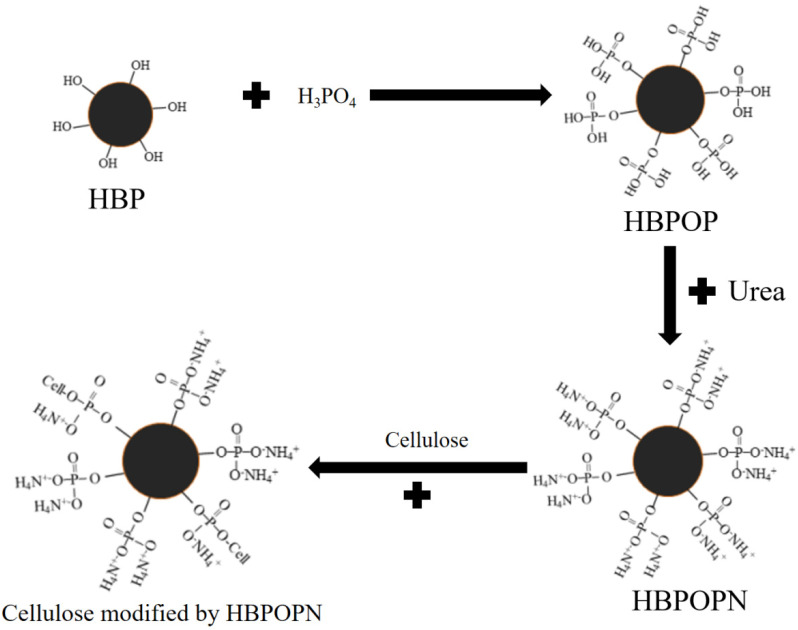
HBPOP-modified cellulose reaction process.

Ling and Guo [41] successfully grafted NH_4_^+^ onto cellulose by mixing cellulose with HBPOPN, resulting in the flame retardancy of cotton fabrics. When 28.1% of HBPOPN was added, the LOI of the cellulose fabric was increased to 42%, and the residual carbon rate was about 35%. The modification process is shown in Figure 5.

**Figure 6:**
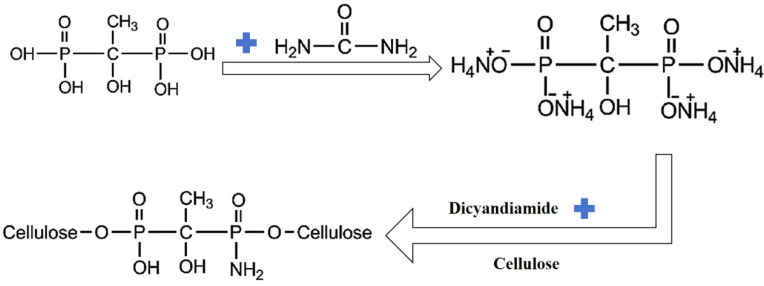
Chemical synthesis of AHEDPA-modified cellulose.

Lu [42] prepared 1-ammonium diphosphonate (AHEDPA) by mixing 1-hydroxyethylidene-1,1-diphosphonic acid (HEDPA) with urea as shown in Figure 6. By adding 20.11% of AHEDPA, the flame retardancy of the cellulosic fabrics was significantly improved, the LOI increased from 18.4% to 41.5%, the residual carbon rate increased from about 10% to about 45% at 600 °C and the residual residue rate measured by CCT increased from 1.3% to 38.9%.

**Figure 7:**
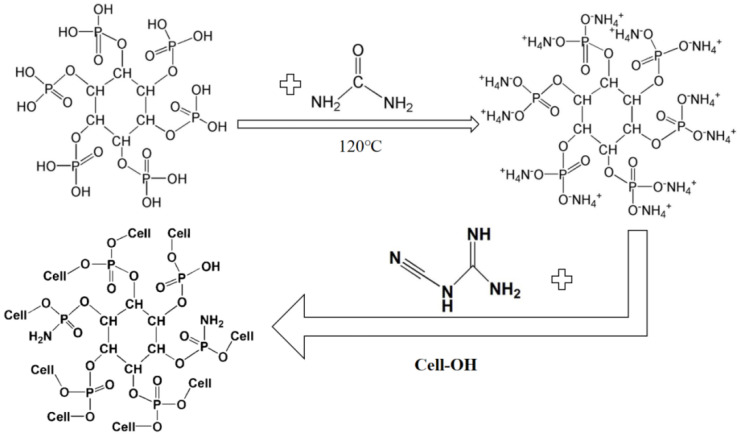
Preparation process of APA-modified cellulose.

Feng [43] prepared ammonium phytate (APA) by mixing phytic acid (PA) with urea. In cellulose fabrics mixed with APA, it was observed that the cation NH_4_^+^ in APA reacts with the -OH of cellulose to produce cellulose fabrics with flame-retardant properties. It was found that the control fabric had an LOI of 17.8% and a residual charcoal rate of about 0.8% at 600 °C. In contrast, the APA-treated cellulosic fabrics had an LOI of 36.1% and a residual carbon rate of about 40% at 600 °C. The results were consistent with those of the AHEDPA-treated cellulosic fabrics. The chemistry of the modification process is shown in Figure 7.

Huang [44] used the reaction of itaconic acid (IA) and DOPO to generate a novel phosphorus-containing flame-retardant-modified DOPO-IA, which was reacted with CNF to successfully prepare cellulose composite aerogels (CNF-DI-x). Compared with the pure cellulose aerogel, the thermal stability, mechanical properties and flame-retardant properties of the DOPO-IA-modified cellulose aerogel were significantly improved. At 700 °C, the residual carbon rate of CNF-DI-3 was 19.8%, which was 187% higher than that of CNF. The pHRR and THR of CNF-DI-3 were reduced by 67% and 60%, respectively, compared with CNF.

**Figure 8:**
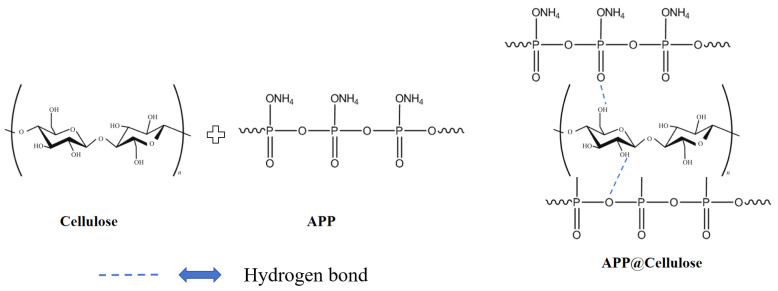
Schematic diagram of the preparation of cellulose fabric coated with APP.

Yin [45] prepared flame-retardant-coated cellulose by immersing cellulose cotton fabric in aqueous APP solution, as shown in Figure 8. The amount of residual carbon on the APP-coated cellulose cotton fabric was significantly increased to 40%, and the breakage length was reduced to 28 cm as measured by a vertical combustion tester.

Although phosphorylated cellulose (e.g., Ghanadpour’s study) can achieve self-extinguishing properties, the literature points to a 20–30% decrease in tensile strength (due to hydroxyl substitution leading to disruption of the hydrogen bonding network), no relevant reinforcement strategies (e.g., nanocellulose reinforcement) have been proposed, and phosphorus-based flame retardants (e.g., APP) may lead to eutrophication of the water column through leaching. Additionally, the study did not conduct a life cycle assessment (LCA) or ecotoxicity tests (e.g., algal growth inhibition experiments). The phosphorylation reaction (e.g., urea/ammonium phosphate system) is highly dependent on cellulose crystallinity (e.g., Noguchi et al.’s study), but it does not model the quantitative relationship between process parameters (temperature, pH) and flame-retardant efficiency, and the process reliability is insufficient.

### 2.4. Sulphur Flame Retardant Modification

Studies have shown that certain modifications of cellulose with ammonium sulphate can also confer certain flame retardancy to NCF. The presence of ammonium sulphate will release NH_3_, SO_2_ and other non-combustible gases, dilute the O_2_ concentration, and at the same time generate sulphuric acid and promote the dehydration of the material carbonisation, thus achieving the effect of flame retardancy [46]. Li [47] synthesised low-eutectic solvents (DES) from sulphamic acid and urea and prepared ASNF20, ASNF30, ASNF40, ASNF50 and ASNF60. The introduced ammonium sulphate salt can provide a flame-retardant effect by promoting the dehydration charring of the material to form a stable char layer. Zhu [48] conducted a similar study and successfully prepared good self-extinguishing and flame-retardant cellulose.

Emilitri and Manfredi [49] synergistically modified cellulose (SS-PAMAM) via 2,2-bis (acrylamido) acetic acid (BAAA) with L-cystine. It was found that SS-PAMAM had 13% residual char at 600 °C, whereas the untreated fabric burned completely in a very short time without forming any residue.

The combustion of sulphur-based compounds (e.g., ammonium sulphate) releases SO_2_ (e.g., Li et al. study), which may exacerbate the risk of asphyxiation in fires, and the difference in smoke toxicity between sulphur-based and other systems (e.g., phosphorus-nitrogen synergism) was not compared in the study. Sulphur-based flame retardants have low LOI increases (e.g., only from 18% to 23% for ASNF60) and insignificant residual carbon increases (e.g., only 13% in Emilitri’s study), which have limited modification effects, and the studies have not elucidated their competitiveness in cellulosic flame-retardant systems.

### 2.5. Inorganic Flame Retardant Modification

The inorganic flame retardant, with its good thermal stability, non-volatility, and less smoke, is a type of environmentally friendly flame retardant. Yang [50] prepared flame-retardant CNF/MoS_2_ aerogels by wrapping cellulose nanofibrils (CNF) using chemical cross-linking of ultrathin MoS_2_ nanosheets. Its LOI was 34.7%, with excellent flame-retardant and self-extinguishing properties.

Yuan [51] prepared a cellulose/SiO_2_ composite aerogel by reacting tetraethyl orthosilicate with cellulose in a different way, and when the content of SiO_2_ was increased, its rate of heat release was decreased, and the composite aerogel appeared to be self-extinguishing when the content of SiO_2_ reached 33.6%. WU [52] prepared cellulose-based composite foams with good flame-retardant properties by introducing silica into cellulose using a chemical in situ method. Yuan [53] prepared cellulose/SiO_2_ composite aerogels using cotton pulp cellulose as the raw material by a sol–gel two-step method. The results showed that the solid barrier formed by SiO_2_ nanoparticles significantly retarded the decomposition of cellulose and inhibited the heat release during combustion, resulting in composite aerogels with excellent resistance to thermal oxidation, thermal stability and flame retardancy.

Amalie [54] used montmorillonite (MMT) and cellulose for blending and found that the addition of MMT significantly improved the heat resistance of the composites, which maintained shape stability at 800 °C, and the flame-retardant properties were also improved.

Liu [55] prepared an NC/MTM organic–inorganic hybrid system. The MTM composite film containing a mass fraction of 50% had a tensile strength of up to 124 MPa, a Young’s modulus of 8.7 GPa, and showed good self-extinguishing properties. Xu [56] prepared CNF/MTM nanocomposite membranes containing carboxylated CNFs (TO-CNFs) by vacuum filtration. When the mass fraction of MTM in the composite film was in the range of 10%~50%, it showed good self-extinguishing property. Zhao [57] successfully prepared ternary CNF/MHNP/RC nanocomposite films with excellent performance using CNFs, MHNPs and regenerated cellulose (RC) as raw materials. The LOI of the composite films increased from 21.5% to 40.2% when the mass fraction of MHNPs in the composite films was increased from 5% to 40%.

Burger [58] mixed dried cellulose pulp with a high concentration of ZnCl_2_ at room temperature, which dissolves cellulose and thus facilitates the nanofibrillation of cellulose pulp. When the mass fraction of ZnCl_2_ in the NC composite film was 33%, the composite film was self-extinguishing after ignition, with only 10% mass loss.

He [59] prepared Al_2_O_3_-SiO_2_/cellulose composite aerogels with good thermal insulation and flame-retardant properties using microcrystalline cellulose as the raw material and Al_2_O_3_-SiO_2_ sol as the flame retardant. The study showed that the maximum decomposition temperature of 205 °C and residual carbon amount of 35.2% were achieved when the volume ratio of Al_2_O_3_-SiO_2_ sol to microcrystalline cellulose solution was 2:10. The ignition time was 11 s and the heat release rate was small, showing better flame retardancy and flame generation inhibition.

Liu [60] used a carboxymethylation reaction to introduce carboxyl groups and metal ions into cellulose to endow it with flame retardancy. It was shown that the LOI value of modified cellulose fibres increased from 17.8% to 35.3%. The pHRR and THRT decreased by 70.1% and 49.4%, respectively, and the densification of the char layer after combustion was improved.

Hu [61] prepared aluminium-doped carboxymethyl cellulose aerogels with flame retardancy that can be rated at the V-0 level. He [62] made AH-NP/CNF composite aerogels with Al(OH)_3_ nanoparticles (AH-NPs) as additives. The composite cellulose aerogel has good flame-retardant properties and self-extinguishes immediately after fire extinguishing, and its pHRR and THR are significantly reduced. Yuan [63] introduced Al(OH)_3_ nanoparticles into cellulose aerogels. During combustion, Al(OH)_3_ in the aerogel decomposed into alumina and released H_2_O, and the high-heat-absorption property of water could reduce the temperature and dilute the combustible gases, which finally inhibited the combustion of the sample. In addition, Al(OH)_3_ nanoparticles formed a protective layer around the cellulose, which further protected the cellulose from burning. The results showed that the THR of the cellulose/Al(OH)_3_ composite aerogel decreased from 11.3 kJ/g to 1.7 kJ/g and the residual carbon increased from 14% to 60% compared to the single-phase cellulose aerogel, indicating that the introduction of Al(OH)_3_ greatly improved the flame retardancy of the cellulose aerogel.

Nabipour [64] prepared zeolite imidazolate backbone-8 composite cellulose aerogels using a layer-by-layer assembly method. The results showed that the pHRR and THR values of the pure cellulose aerogel were 140.4 kW/m^2^ and 13.0 MJ/m^2^, respectively. The pHRR and THR values of the composites decreased gradually with the increase in the mass fraction of zeolite imidazolate backbone-8. The composite cellulose aerogel assembled with three layers of zeolite imidazolate backbone-8 showed the best flame retardancy.

SiO_2_ needs to be added at more than 30% to achieve self-extinguishing (e.g., Yuan’s study), but it will lead to a 40% increase in the density of the material and a rise in brittleness, and research has not explored the efficient flame-retardant strategy of nanosized SiO_2_ (e.g., mesoporous structure) with a low additive amount. Physical adsorption between inorganic fillers (e.g., MoS_2_ nanosheets) and cellulose is dominant, which may be susceptible to interfacial peeling due to differences in thermal expansion coefficients in long-term use (e.g., Yang et al. did not provide cyclic thermal shock test data). Also, most of these studies focus on the flame retardancy of cellulose and do not fully utilise the multi-functional potential of the inorganic phase (e.g., UV shielding by ZnO, smoke suppression by Al(OH)_3_).

### 2.6. Hybrid Co-Modification

#### 2.6.1. N/P-Based Synergistic Modification

Zheng [65] reacted ethylenediamine tetramethylene phosphonic acid (EDTMPA) with urea to produce AEDTMPA, which was added to cellulose fabrics to finally prepare flame-retardant cellulose containing P-O-C covalent bonds. Compared with the unmodified cellulose fabric, the LOI of the modified sample increased from 20% to 43.6%, and the residual carbon rate increased from 8% to 43.4%. The untreated cellulosic fabrics burned completely, whereas the AEDTMPA-treated textiles had a breakage length of only 3.5 cm in the vertical burn test.

Wan [66] prepared aminomethane penta(methylphosphonic acid) ammonium salt (ATPMPA) by the chemical reaction of tris (hydroxymethyl) aminomethane with formaldehyde, H_3_PO_3_, H_3_PO_4_ and urea. When ATPMPA is added to cellulosic cotton fabrics, NH_4_^+^ in ATPMPA reacts with the -OH of cellulose to produce cotton fabrics with flame-retardant properties. Similar studies were conducted by Tian, Li, Huang and Zhang [67,68,69,70], which showed an increase in LOI, residual char rate, and residual residue rate as determined by conical calorimetry, as well as a decrease in the [CO_2_]/[CO] ratio, which suggests that these compounds have excellent flame-retardant properties.

**Figure 9:**
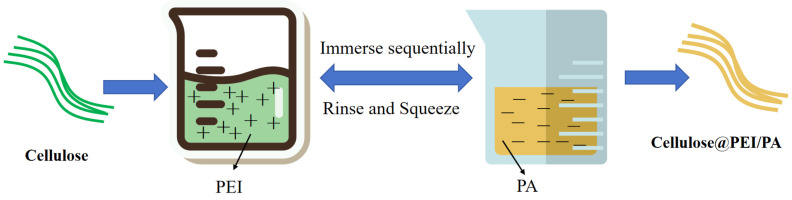
Preparation of flame-retardant PEI/PA/cellulose fabrics.

Zhang [71] prepared cellulosic fabrics with cationic polyethyleneimine (PEI) and anionic phytate (PA) bilayer structures by a layer-by-layer deposition method. The layer-by-layer deposition process is shown in Figure 9. Compared to the control (18.5% LOI and 16.3% residual carbon), the treated cellulosic cotton fabrics showed an increase in LOI value up to 37% and residual carbon up to 35%.

Although these studies proposed the synergistic flame-retardant effect of N/P, they did not clarify the contribution ratio of nitrogen (e.g., NH_3_ release) and phosphorus (e.g., PO radical generation) at different stages of combustion by in situ analysis (e.g., TG-FTIR), which would lead to the lack of a scientific basis for the subsequent optimisation of the formulations. Meanwhile, most of the studies only tested the initial flame retardancy (e.g., vertical self-extinguishing combustion) but did not simulate the effects of washing, friction or UV aging on the stability of the N/P bonds in actual use (e.g., Zheng’s study did not provide data on water washing resistance). Layer deposition methods (e.g., PEI/PA coatings) require multiple dipping–drying cycles (5–10), which is time-consuming and energy-intensive, and the study did not explore the feasibility of a continuous process (e.g., spraying or roller coating) for practical applications.

In addition to these N/P-based flame retardants, there are a number of new advances in the current research on N/P-based flame retardants that may also be expected to be applied in the cellulose field in the future. For example, Konstantinova [72] condensed 4-formylphenoxyphenoxycyclic triphosphazene with malonic acid to obtain 4-(β-carboxyvinyl)phenoxyphenoxycyclic triphosphazene (CPPP). The structure was investigated using 31P, 1H and 13C NMR spectroscopy and MALDI-TOF mass spectrometry, and the thermal properties were determined using DSC and TGA methods. Konstantinova utilised CPPP to cure the epoxy resin DER-331 and assessed the conversion of the epoxy groups using infrared spectroscopy, with optical interferometry showing that CPPP was compatible with the epoxy resin over the temperature range of 80 to 130 °C. CPPP was also used in the curing of the epoxy resin DER-331. Optical interferometry showed that CPPP had good compatibility with epoxy resins in the temperature range of 80 to 130 °C. The cured epoxy compositions were fire-resistant, as porous coke was formed during combustion, and successfully passed the UL-94 vertical combustion test, as well as being highly heat-resistant and thermally stable (decomposition onset temperature of about 300 °C, glass transition temperature of 230 °C). With low water absorption, high resistance to fresh and salt water, good fire resistance, and high bonding strength (11 ± 0.2 MPa) to steel and aluminium, the composition is expected to be used as an adhesive component for bonding parts in the shipbuilding, automotive, aerospace and radio-electronics industries.

Терехoв [73] added isomethyltetrahydrophthalic anhydride and a novel epoxide containing aryloxycyclotriphosphine to D.E.R.-330 resin and studied the combustion resistance of the epoxy compositions, as well as thermogravimetric analysis and microstructural studies of the coke residue formed during the combustion process. The results show that an increase in the content of phosphazene in the cured compositions significantly increases their combustion resistance, which is associated with an increase in the amount of porous coke residue formed during combustion, which serves as a barrier to flame propagation and heat transfer from the flame to the sample, as well as with an increase in the size of the pores formed in the coke residue. The data obtained can be used to create strong, combustion-resistant composites for microelectronics, aircraft manufacturing and other industrial applications.

Yao [74] synthesised a novel reactive flame retardant, 1,4-bis-DOPO-(1,4-bis((1-hydroxy-2-ethyl-ethyl)amino)methyl)benzene) (PABD), which contains the elements phosphorus (P) and nitrogen (N), as well as pendant chains, and produced a high-performance flame-retardant polyurethane elastomer (PUE-PABD) using PABD. Flame retardancy testing demonstrated that PUE-PABD15 has excellent flame retardancy and is UL-94 V-0 rated with a limiting oxygen index (LOI) of 29.1%. Compared with unmodified PUE, PUEPABD15 has 82.6% and 49.5% lower peak and total heat release rates, respectively, and 70.7% lower peak and total smoke production. Dynamic mechanical analysis showed that the maximum tan delta value was increased from 0.39 to 0.74, and the effective damping temperature range was expanded from 29.3 degrees Celsius to 54.1 degrees Celsius. In addition, the incorporation of 15.0 wt.% PABD increased the tensile strength of PUE by 108.5%, which was attributed to the enhanced hydrogen bonding and pi–pi stacking by the benzene ring group.

These flame retardants act in the same gas phase and coalescence through the synergistic action of nitrogen (N) and phosphorus (P), e.g., Yao’s [74] PABD captures flammable radicals by releasing phosphorus-containing radicals (e.g., -PO-), while the decomposition of nitrogen generates NH_3_ to dilute the oxygen concentration, which significantly reduces the rate of heat release (THR reduced by 49.5%) and smoke production (reduced by 70.7%), and this dual mechanism is superior to single-action boron or sulphur flame retardants. At the same time, they can not only enhance the flame retardancy of the material but also the mechanical properties and environmental resistance of the material, as well as having the advantages of environmental protection, low toxicity and a low additive amount to realise the advantages of efficient flame retardancy, with a wide range of prospects for application, and they are expected to show their unique advantages in cellulose and other composite materials.

#### 2.6.2. Synergistic Modification with N/P/Si Groups

The preparation of N/P/Si-based flame-retardant cellulosic fabrics can be achieved by a two-step process, whereby cotton fabrics are first treated with 3-mercaptopropyltriethoxysilane (MPTES), followed by dimethyl -[1,3,5-(3,5-triacryloyloxyhexahydro) triazinyl]-3-oxypropylphosphonate (DHTP). In the reaction with MPTES, the ethoxy group at one end of MPTES reacts with the hydroxyl group on the surface of cellulose to form a covalent bond between cellulose and MPTES through O-Si bonding (Sun [75]). In the reaction with DHTP, sulphydryl groups on the MPTES-treated cellulosic cotton fabric react with DHTP to produce a halogen-free, organophosphorus-based, flame-retardant cellulosic cotton fabric (Yoshioka-Tarver et al. [76]; Xu et al. [77,78]). It was found that the LOI of the flame-retardant cellulosic cotton fabrics increased from 18.3% to 34%, and the residual char at 600 °C increased from 10% to 43% compared to the control group. The synthesis of DHTP is described in other studies (Weil [79,80]).

**Figure 10:**
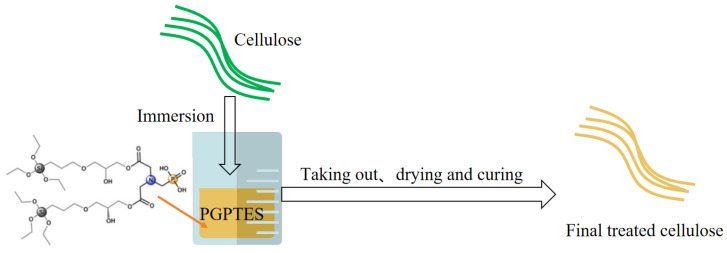
Preparation of PGPTES/cellulose fabrics.

Castellano [20] used (3-glycidoxypropyl) triethoxysilane-modified N-(phosphonomethyl) iminodiacetic acid (PGPTES) for the modification of cellulosic fabrics. It was found that the residual char, CO_2_/CO ratio and residual char at 600 °C were 38%, 20 and 26%, respectively, with a maximum char length of 5 cm for cellulosic fabrics spiked with 25.2% PGPTES. The whole preparation process with PGPTES/cellulose fabrics is shown in Figure 10.

Zhao [81] prepared H-DPTA by reacting 3- triethoxymethylsilyl propylamine with chlorodiphenyl phosphine (Ph_2_PCl). Subsequently, flame-retardant cellulose cotton fabrics were obtained by treating cellulose fabrics with H-DPTA. The TG and LOI tests showed that the residual carbon rate at 600 °C of the treated samples increased from 15% to 42%, and the LOI increased from 18.4% to 25.4%.

In conclusion, DHTP, PGPTES and DPTA are non-polymeric organic compounds based on nitrogen (N), phosphorus (P) and silicon (Si), and they are able to improve the flame-retardant properties of cotton fabrics.

The synthesis process of silane reagents (e.g., MPTES) involves toxic solvents (e.g., toluene) and some silanes (e.g., chlorosilanes) may release HCl, but the ecological impacts of their production and use have not been evaluated in the study, which is an environmental risk. Moreover, these modification methods require multi-step chemical reactions (e.g., MPTES pretreatment → DHTP grafting), harsh reaction conditions (e.g., anhydrous environments) and are susceptible to side reactions (e.g., self-condensation of silanes) when scaled up industrially, resulting in decreased yields. The silane coupling agent is expensive (about 50–100/kg), which is more than 10 times that of traditional flame retardants (e.g., APP, 50–100/kg), and research has not proved its economic feasibility through cost-performance modelling.

#### 2.6.3. Organic/Inorganic Synergistic Flame Retardancy

**Figure 11:**
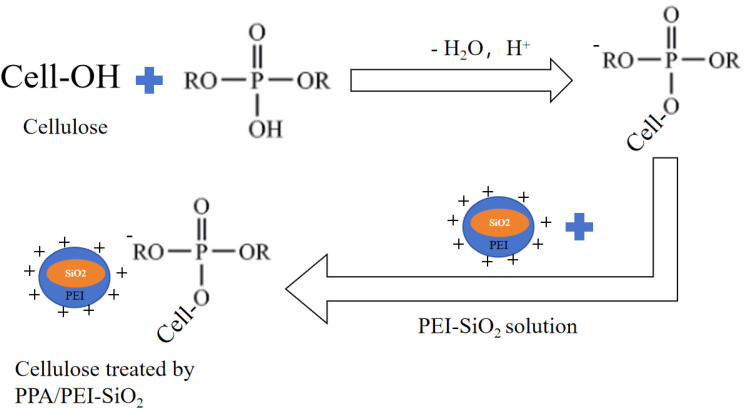
Preparation of PPA/PEI-SiO_2_ cellulose fabrics.

Lessan [82] placed cellulose fabrics into a mixed dispersion of sodium hypophosphite (SHP), maleic acid (MA), triethanolamine (TEA) and titanium dioxide nanoparticles (TiO_2_), producing flame-retardant cellulose fabrics by drying, curing and washing. The LOI test showed that the treated cellulose fabrics had an LOI of 22.7%, which was 21% higher, and the residual carbon was 27%, indicating that the treated cellulose fabrics were flame-retardant. Li [83] used a layer-by-layer deposition method to immerse cellulose fabrics in a solution of negatively charged polyphosphoric acid (PPA), which was then immersed in an aqueous dispersion consisting of a mixture of PEI and SiO_2_, which then formed a layer of flame-retardant coating on the cellulose fabrics. The chemical reaction for the formation of the PPA/PEI-SiO_2_ coating on cellulosic cotton fabrics is shown in Figure 11.

Zhang [84] prepared another flame-retardant-deposited cellulose fabric. Firstly, the cellulose fabric was placed into a positively charged chitosan solution. Then, the dried cellulose fabric was immersed in phytic acid solution, and the dried cellulose fabric was immersed in barium ion (Ba^2+^) solution. Finally, the cellulose fabric co-coated by chitosan, phytic acid and barium ions was obtained. Cheng [85] prepared a two-component solution coating consisting of PA and SiO_2_ for use in cellulosic fabrics. The phytic acid/silica solution was first prepared by a chemical reaction between ethyl orthosilicate (TEOS), ethanol and phytic acid, and then the cellulosic fabric was immersed in it, which could be obtained by drying and curing.

Cellulose fabrics were prepared by layer-by-layer deposition [82,83,84,85]. All of them showed the phenomenon of self-extinguishing from fire, and all of them also increased their residual carbon rate at 600 °C.

Metal–organic frameworks (MOFs) can be constructed by connecting inorganic nodes and organic linkers with diverse structures, large aspect ratios, and highly tuneable large pore sizes. Nabipour [64] prepared ZIF-8@cellulose composite aerogels by introducing zeolite imidazolium salt framework-8 (ZIF-8) onto cellulose surfaces via an in situ growth method. The thermal stability and flame retardancy of the modified cellulose aerogels were substantially improved, and the pHRR and THR values were significantly reduced.

Guo [86] synthesised a novel P/N-containing flame retardant (TPM-PAT), followed by secondary modification of the TPMPAT/CNF aerogel with polydimethylsiloxane (PDMS). The PDMS-TPMPAT/CNF aerogel showed better thermal stability and a higher LOI. Yan [87] developed anisotropic cellulose (CNF-Si-T) aerogels by modifying cellulose using methyl trimethoxysilane (MTMS) and p-toluenesulphonic acid (TsOH). CNF-Si-T showed excellent flame-retardant properties, with LOIs as high as 42.6%~51.0%. The flame-retardant mechanism of CNF-Si-T aerogels mainly involves two parts. On the one hand, TsOH promotes the dehydration of cellulose into char at low temperatures, and this char layer prevents the transfer of heat and oxygen inside and outside, slowing down combustion. On the other hand, during cellulose degradation, Si-H_3_ pyrolysis in MTMS forms a uniform and dense Si-O-Si interfacial barrier. When combustion occurs, the rapid formation of the SiO_2_ barrier slows combustion by blocking heat, oxygen and combustible volatiles.

Organic/inorganic phases are prone to phase separation due to polarity differences (e.g., TiO_2_ agglomerates on cellulose surfaces), but these studies did not use surface modification techniques (e.g., silanisation of SiO_2_) or nanodispersion techniques (e.g., ultrasound-assisted) to improve their compatibility. Moreover, inorganic fillers require high loading (e.g., SiO_2_ addition >30%) to be effective flame retardants, which leads to an increase in the density of the material (e.g., a 50% increase in the density of the composite aerogel) and a loss of the lightweight advantage of the material. Similar to the inorganic additive modifications mentioned above, these studies also focus on flame-retardant properties and do not explore the additional functions of the inorganic phase (e.g., photocatalytic self-cleaning of TiO_2_, sound insulation of SiO_2_), which limits the multi-scenario applicability of the composites.

## 3. Application of Flame-Retardant Cellulose in Composite Materials

With the development of society, people pay more and more attention to energy saving, emission reduction and environmental protection, and bio-based flame-retardant composites are the trend of the times; therefore, this section conducts a systematic review of bio-based flame-retardant materials, and quite a number of flame-retardant cellulose composites are also mentioned in the previous section where various types of flame-retardant cellulose modification methods are reviewed, for example, flame-retardant cellulose aerogel materials (Cheng [15], Qin [16], Yuan [63], etc.), flame-retardant cellulose foams (Wu [51], etc.), flame-retardant cellulose membranes/films (Leaaan [82], etc.), and flame-retardant cellulose fabrics (Castellano [20], Lu [42], Feng [43], Zhang [71], etc.), which will not be repeated below, as well as different forms of cellulose, types of polymers used and the flame-retardant properties of specific cellulose composites. Polymer types and flame-retardant properties are specified in Table 1 at the end of this section.

### 3.1. Flame-Retardant Cellulose Aerogel Composites

Cellulose aerogel (CNF) is usually physically blended to improve the toughness of polymers, but due to the large aspect ratio and specific surface area of CNF itself, it is easy to aggregate in polymers; therefore, different preparation or modification methods are usually used to introduce CNF into polymer materials to realise the performance enhancement.

Suo [40] prepared CNC@DPP flame retardants and added them to epoxy resin (EP). When the EP composites burned, DPP decomposed at high temperatures to generate oxygenated acids such as phosphoric acid, which promoted the dehydration of CNC and EP into charcoal, forming a dense and continuous char layer to inhibit heat and heat propagation. Furthermore, the addition of CNC@DPP does not have much effect on the mechanical properties of EP composites.

Gou [88] used phosphorylated cellulose to prepare a complex gel polymer electrolyte (GPE) for lithium ion batteries, and the prepared GPE showed good tensile strength and self-extinguishing properties. Similarly, Aziam [89] utilised P-NC as an organic filler for solid polymer electrolytes, improving the mechanical properties and thermal stability of the electrolytes. This is the first time that PCNFs have been used as reinforcing fillers in solid polymer electrolytes for lithium batteries.

Yan [90] prepared cellulose nanofiber/carboxylated multi-walled carbon nanotube aerogels (CCA80) and impregnated them with a mixture of epoxy resin (EP) and a synergistic flame retardant (FR) of hexaphenoxycyclic triphosphazene and poly (piperazinylmethylphosphonate) pentaerythritol ester to obtain EP/CCA80/FR composites. Compared with pure EP, EP/CCA80/6 wt.% FR showed a 38.9% increase in ultimate oxygen index, and a 30.61% and 39.47% reduction in total heat release and carbon dioxide generation rates, respectively, to meet the UL-94 V-0 standard. In addition, it had a thermal conductivity of 1.06 W·m^−1^·K^−1^ and 1.0 kW·m^−2^, which was 457.9% higher than pure EP, while maintaining excellent electrical insulation. Under 1.0 kW·m^−2^ irradiation for 2 h, its surface temperature remained at 94.5 °C, showing good light and heat conversion ability and anti-photo-bleaching ability.

Yan [91] fabricated an epoxy resin (EP) nanocomposite EP/CCA80 with excellent photothermoelectric conversion properties by embedding vertically aligned aerogels, consisting of cellulose nanofibers (CNFs) and carboxylated multi-walled carbon nanotubes (CMWNTs), into a transparent epoxy resin (EP) matrix. The EP/CCA80 composite has a wide light absorption range from 200 nm to 2500 nm and excellent photothermal properties. EP/CCA80 achieves a remarkable stabilisation temperature of 93.2 °C and a photothermal conversion efficiency of 54.35% under a light irradiation of 1.0 kW·m^−2^, with a content of CMWCNTs of only 0.65 wt.%. In addition, the EP/CCA80 composite, in combination with a thermoelectric (TE) device, produces significant temperature differences and voltage outputs of up to 25.3 °C and 160.29 mV (1.0 kW·m^−2^), respectively, which can power a small fan rotating at 193 min^−1^.

Du [92] synthesised two-dimensional (2D) layered black phosphorus (BP) nanosheets from black phosphorus crystals by ultrasound-assisted liquid-phase exfoliation with excellent photothermal effects. Then, novel form-stable organic phase change material (PCM) composites (CBPCMs) were prepared by impregnating n-octacosane into cellulose nanofiber (CNF)/BP hybrid aerogels. The porous aerogel can fully support n-eicosane and prevent liquid leakage. Differential scanning calorimetry (DSC) analysis showed that the CBPCMs synthesised on the basis of CNF/BP hybrid aerogels had very high n-alkane loading capacity and heat storage density (247.0–251.6 J·g^−1^). The incorporation of BP nanosheets into the aerogels significantly increased the thermal conductivity (by 89.0%) and solar–thermal conversion and storage efficiency (up to 87.6%) of the CBPCMs. In addition, with the increase in BP nanosheet content in the aerogel, the heat release rate and total heat release of CBPCMs decreased dramatically, while the LOI value and charring rate increased, which indicated that the flame-retardant properties of the PCM composites were significantly improved.

### 3.2. Flame-Retardant Cellulose PP Composites

Li [93] modified wheatgrass cellulose using chitosan under alkaline conditions and filled modified wheatgrass cellulose as filler into polypropylene (PP). It was found that when the modified wheatgrass cellulose was added at 25%, the PP composite had the highest crystallinity, and the maximum exothermic rate was 190.6 kW/m^2^. Then, it decreased by 35.1% year-on-year, and the flame-retardant effect was obvious. Thanh [94] investigated the preparation of reinforced PP composites by extrusion injection moulding using short woven flax cellulose (SWF), short basalt fibre (BF) and rice husk powder (RHP) as fillers. The pHRR of 25SWF/20BF/PP.6MAPP-18RHP composites was reduced by 80.95% and THR by 23.84% compared to pure PP. In addition, the amount of CO and CO_2_ released from the composites during combustion was reduced by 68% and 67%, respectively, and the fire growth index (FGI) was improved by 81.34%. Gairola [95] used borax as a modifier for cellulose and subsequently added the modified cellulose to PP to prepare flame-retardant composites. The PP composites were found to be more thermally stable and flame-retardant as compared to pure PP and showed an increase in LOI by 22.01% and a decrease in pHRR by 22.29%. This is mainly due to the formation of a dense fibrous structure in the PP composites during combustion, which acts as a protective barrier to impede the transfer of heat and oxygen, thus improving the flame retardancy.

Kang [96] deposited APP and PEI onto the cellulose surface by a layer-by-layer self-assembly technique. Then, 30% modified cellulose was added to the PP matrix, the residual carbon of PP composites increased from 3.10% (PP) to 15.77% (PP/modified cellulose) and its LOI increased from 19.4% (unmodified) to 23.2%. This is mainly due to the APP/PEI system deposited on the surface of the cellulose inhibiting its combustion and the constructed flame-retardant network limiting the combustion of the polypropylene matrix. Yu [97] introduced tannic acid (TA)-Fe^3+^ complexes and halloysite nanotubes (HNTs) onto the cellulose surface and prepared bamboo cellulose/polypropylene composites by a hot pressing process. The PP composites had higher thermal stability and residual carbon rate in TG tests compared to pure PP. Moreover, due to the flame-retardant effect of the TA-Fe^3+^@HNTs layer in the gas and condensed phases, the THR and TSP of the PP composites were reduced by 23.75% and 32.44%, respectively, which exhibited significant flame-retardant properties. Kim [98] prepared an EPS cellulose/PP composite by adsorbing an extracellular polymer (EPS) onto the surface of cellulose, which was then blended with PP. TG tests showed that the EPS-cellulose/PP composite had a higher residual carbon, and the EPS-cellulose/PP composite appeared to be self-extinguishing during vertical combustion.

Liu [99] modified microcrystalline cellulose (Mcc) with stearic acid (SA) as a modifier and prepared Mcc/PP composites by extrusion moulding. The best mechanical properties of PP composites were obtained when 9% of SA-Mcc was added, at which time the crystallisation temperature and maximum degradation temperature of PP composites were increased, and the thermal stability of the material was improved. Zheng [100] prepared cellulose/PP composites (PP/HECPM) by extrusion–hot press moulding using the chemical introduction of phosphoric acid and melamine groups on the cellulose structure. When HECPM was added at 30%, the residual carbon of PP/HECPM at 600 °C was 7.33, which was significantly higher than that of PP (0%), and the LOI value of PP/HECPM increased from 17% to 26.4%, indicating that PP/HECPM had higher thermal stability and flame retardancy.

### 3.3. Flame-Retardant Cellulose PLA Composites

Wang [101] synergistically modified microcrystalline cellulose with phosphorylation and phosphoramidification to successfully prepare phenylphosphoramidated microcrystalline cellulose (PhPNMCC), which was then blended with PLA to obtain flame-retardant PLA composites. After adding 4% of PhPNMCC, the PLA composites reached UL-94 V-0 level. It is shown that PhPNMCC can improve the flame retardancy of PLA and inhibit the melting droplet effect, which is mainly attributed to the phosphorus–oxygen radicals in PhPNMCC inhibiting the decomposition rate of PLA, and the stabilised phosphorus-rich residue containing a synergistic barrier effect on the condensed phase.

Yang [102] used sulphamate-based solvent (SDES) to modify straw cellulose and subsequently blended with PLA to prepare DS/PLA composites. It was found that the LOI of DS/PLA composites reached 36.53%, the residual carbon was enhanced from 7.8% to 38.4% and the tensile modulus was increased by 69.5%.

### 3.4. Flame-Retardant Cellulose Epoxy Composites

Meng [103] synthesised N,P-containing cellulose composites (NPCNCs) by the ice bath polymerisation technique, which were subsequently added to EP to prepare flame-retardant EP/NPCNC materials with excellent properties. The LOI of EP composites increased from 23.5% (EP) to 27.6% with the addition of 6% NPCNCs. Compared with EP, the THR, pHRR, TSP and SPR of EP/6NPCNCs were reduced by 27.27%, 43.34%, 70.21% and 66.67%, respectively.

Adil [104] successfully prepared flame-retardant natural fibre-reinforced green composites (NFRGCs) by combining cellulose filaments (CLFs) with vanillin-derived epoxy resin (VDE) using hand lay-up and compression moulding techniques. Compared with the pure VDE resin, the NFRGC material can achieve a V-0 combustion rating, indicating its excellent flame-retardant properties. In addition, the green composite exhibits excellent hydrophobicity, with a water contact angle of 104.2°.

### 3.5. Flame-Retardant Cellulose Polyurethane Composites

Huang [105] mixed phosphorylated cellulose (PMFC) with waterborne polyurethane (WPU) to prepare flame-retardant composites. It was found that the flame retardancy and abrasion resistance of the composites were substantially improved by adding an appropriate amount of PMFC to the WPU coatings.

When cellulose is composited with epoxy resin, the difference in polarity can lead to a decrease in interfacial bond strength (e.g., 15% reduction in tensile modulus of epoxy composites in Suo et al.’s study), but they did not use coupling agents (e.g., silane coupling agents) or surface grafting modifications to improve only material compatibility. These studies (e.g., Adil’s natural fibre-reinforced composites) did not provide flame-retardant dispersion uniformity (e.g., SEM-EDS mapping) or batch stability data (e.g., LOI fluctuation ranges) at the pilot scale, and data for large-scale production are missing. On the other hand, the multi-functional integration of these studies is insufficient, mostly focusing on a single flame-retardant property, without exploring the multi-functional synergies (e.g., integrated flame-retardant–acoustic insulation–lightweight design) of flame-retardant cellulose in composites.

## 4. Conclusions

Cellulose is a green and sustainable material, which is in accordance with the current world development trend. The flame-retardant modification of cellulose can reduce environmental pollution and the release of toxic gases, and cellulose has excellent charcoal-forming properties and is expected to replace commercial charcoal-forming agents. In addition, flame-retardant cellulose also shows good performance and potential in the application of composite materials. For example, the addition of flame-retardant cellulose to polymers such as EP, PP, PLA, etc. not only improves the flame-retardant properties of the materials but also improves their mechanical properties and thermal stability, which helps them to be more promising in aerospace, automotive manufacturing, electronics, electrical appliances and other fields.

Although significant progress has been made in the research of cellulosic flame-retardant materials, current studies have significantly improved their flame-retardant efficiency and functional properties through multi-faceted modification strategies (e.g., boron, phosphorus, and nitrogen synergism and inorganic composites). However, current research still faces the following core challenges:

Technological voids: Existing solutions rely on trial-and-error methods and lack quantitative design principles for the interaction between the cellulose structure and flame retardants. In the future, in situ characterisation techniques (e.g., in situ X-ray diffraction, dynamic thermo-mechanical analysis) are needed to build closed-loop structure–property–mechanism models to guide the molecular design of highly effective flame retardants.

Sustainability fault: Most studies focus only on the short-term improvement of flame-retardant performance, ignoring the full life cycle impact. Therefore, it is necessary to introduce a life cycle assessment (LCA) framework to quantify the carbon emissions and ecotoxicity of flame retardants at the synthesis, use and disposal stages, and to prioritise the development of bio-based flame retardants (e.g., lignin derivatives, chitosan–phytanic acid complexes).

Industrialisation bottlenecks: Conversion from laboratory results to large-scale production is limited by energy-intensive processes (e.g., layer deposition) and interfacial compatibility deficiencies. There is a need to develop continuous production technologies (e.g., microreactor synthesis of flame retardants, integrated roll coating–hot pressing moulding) and to design low-cost coupling agents (e.g., bio-based silanes) to optimise the interfacial bonding between cellulose and the polymer matrix.

Therefore, future research directions in the field of flame-retardant cellulose can focus on the innovation of green flame retardants, such as using agricultural waste (e.g., rice husk to extract SiO_2_) or marine biomass (e.g., chitin nanocrystals) to develop renewable flame retardants and reduce the reliance on petrochemical feedstocks, or designing smart coatings through dynamic covalent bonds (e.g., disulphide bonding and boronate bonding) to autonomously repair the flame retardant in the case of a fire warning or after a mechanical injury. At the same time, it is also necessary to promote the intelligent and multi-functional integration of research, which can use machine learning to screen combinations of flame-retardant elements (e.g., N/P/Si ratios), predict the thermal degradation behaviour and toxicity by-products, accelerate the development of high-performance flame retardant, or composite flame-retardant cellulose with electrically conductive materials (e.g., carbon nanotubes, MXene), and develop intelligent fire-resistant materials for real-time monitoring of temperature or smoke; on the other hand, we also need to Focus on breakthroughs in industrialised adaptation technology, explore low-energy process design, use energy-saving technologies such as photoinitiated grafting, plasma surface modification, etc., to replace the traditional high-energy methods (e.g., electron-beam irradiation), and also pay attention to the validation of large-scale production, which can be used to jointly set up a pilot platform for enterprises to validate the continuous production process for flame-retardant wood and composite materials (e.g., impregnation-drying-curing integrated assembly line), and to Formulate industry standards (e.g., flame retardant grade, mechanical properties threshold).

Flame-retardant cellulose has a broad development prospect and will profoundly affect many fields, for example, in the field of green building and transportation, flame-retardant wood composite materials can replace traditional gypsum board or metal fireproofing layer, used for the interior wall of the building or high-speed rail interior, with both lightweight (density reduced by 20–30%) and low-carbon advantages. Cellulose-based flame-retardant foam is used in the thermal insulation layer of battery packs for new energy vehicles to inhibit the spread of thermal runaway and improve safety. In the field of electronics and energy, flame-retardant cellulose aerogel can be used as a lithium ion battery diaphragm, which can form a stable charcoal layer at high temperatures, preventing short-circuits and prolonging the life of the battery. Flexible flame-retardant cellulose film can be used for wearable devices, through the phosphorus–nitrogen synergistic system, to achieve the dual function of fire prevention and electromagnetic shielding. Flame-retardant cellulose will also promote economic recycling and green development, such as the development of biodegradable flame-retardant packaging materials (e.g., cellulose/polylactic acid composite film), reduce plastic pollution, and through closed-loop recycling technology, achieve the regeneration of flame retardants.

In conclusion, flame-retardant cellulose materials are moving from laboratory innovation to industrialised applications, and their future breakthroughs depend on the deep integration of green chemistry, intelligent manufacturing and interdisciplinary collaboration. By overcoming the challenges of interface engineering, full life cycle sustainability and large-scale production, this field is expected to lead the innovation of a new generation of high-performance, multi-functional, fire-resistant materials and provide key technological support for the goal of sustainable development.

## Figures and Tables

**Table 1:** Summary classification table of flame-retardant cellulose and its composites.

Flame-Retardant Cellulose Composites	Cellulose Form	Polymer Matrix	Flame Retardant Type	LOI (%)	UL-94 Rating	Residual Carbon (%)
Zinc borate-modified cellulose aerogel (Cheng [15])	aerogel	/	ZB	/	/	/
In situ synthesis of flame-retardant cellulose aerogels (Qin [16])	aerogel	/	ZB	/	/	/
PGPTES-modified cellulose fabrics (Castellano [20])	woven material	/	PGPTES	/	/	38% (600 °C)
CNC@DPP flame-retardant epoxy composite (Suo [40])	aerogel	EP	cellulose phosphate (CNC@DPP)	/	/	/
AHEDPA-modified cellulose fabric (Lu [42])	woven material	/	AHEDPA	41.5	/	45% (600 °C)
Ammonium phytate-modified cellulose fabric (Feng [43])	woven material	/	APA	36.1	/	40% (600 °C)
Aluminum hydroxide-modified cellulose aerogel (Yuan [63])	aerogel	/	Al(OH)_3_	/	/	60% (Compared to single-phase cellulose aerogels.)
ZIF-8@ cellulose composite aerogel (Nabipour [64])	aerogel	/	ZIF-8	/	/	/
Cellulose cotton fabric with double-layer structure (Zhang [71])	woven material	cotton	PEI and PA	37.0	/	35% (600 °C)
Nano TiO_2_-modified cellulose fabrics (Lessan [82])	woven material	/	TiO_2_ nanometer	22.7	/	27%
PPA/PEI-SiO_2_ cellulose fabric (Li [83])	woven material	/	PPA/PEI-SiO_2_	/	/	/
Chitosan, phytic acid and barium ion coated cellulose fabrics (Zhang [84])	woven material	/	chitosan, phytic acid and barium ions	/	/	/
Phytic acid/silica coated cellulose fabric (Cheng [85])	woven material	/	phytic acid/silica	/	/	/
PDMS-TPMPAT/CNF aerogel (Guo [86])	aerogel	/	PDMS-TPMPAT	/	/	/
Heterogeneous cellulose aerogel (Yan [87])	aerogel	/	MTM and TsOHS	42.6–51	/	/
Phosphorylated cellulose composite gel polymer electrolyte (Gou [88])	aerogel	polymer electrolyte	phosphorylated cellulose	/	/	/
Phosphorylated cellulose solid polymer electrolyte (Aziam [89])	aerogel	solid polymer electrolyte	phosphorylated cellulose	/	/	/
EP/CCA80/FR (Yan [90])	composite material	EP	FR	38.9%	V-0	/
EP/CCA80 (Yan [91])	composite material	EP	/	/	/	/
CBPCMs (Du [92])	composite material	PCM	/	/	/	/
Modified wheatgrass cellulose polypropylene composites (Li [93])	composite material	PP	chitosan-modified cellulose	/	/	/
Short braided flax cellulose polypropylene composites (Thanh [94])	composite material	PP	SWF	/	/	/
Borax-modified cellulose polypropylene composites (Gairola [95])	composite material	PP	ZB	22.01	/	/
APP/PEI coated cellulose polypropylene composite (Kang [96])	composite material	PP	APP/PEI	23.2	/	15.77
Tannic acid-Fe^3+^ complex cellulose polypropylene composites (Yu [97])	composite material	PP	tannic acid–Fe^3+^ complexes	/	/	/
EPS cellulose polypropylene composite (Kyeun [98])	composite material	PP	EPS	/	/	/
Stearic acid-modified cellulose polypropylene composites (Liu [99])	composite material	PP	SA	/	/	/
Phosphoric acid- and melamine-modified cellulose polypropylene composites (Zheng [100])	composite material	PP	phosphoric acid and melamine	26.4	/	7.33% (600 °C)
Phosphorylated and phosphoramidated modified cellulose PLA composites (Wang [101])	composite material	PLA	phosphorylated and phosphoramidated cellulose	/	V-0	/
Deep eutectic solvent-modified straw cellulose PLA composites (Yang [102])	composite material	PLA	SDES	36.53	/	38.4%
Nitrogen- and phosphorus-containing cellulose epoxy composites (Meng [103])	composite material	EP	nitrogen- and phosphorus-containing cellulose	27.6	/	/
Natural fiber reinforced vanillin derived epoxy composites (Adil [104])	composite material	vanillin-derived epoxy resin	natural fiber	/	V-0	/
Phosphorylated cellulose waterborne polyurethane composites (Huang [105])	composite material	WPU	phosphorylated cellulose	/	/	/
